# Poly[(μ_3_-benzene-1,3,5-tricarboxyl­ato-κ^3^ *O* ^1^:*O* ^3^:*O* ^5^)(μ_2_-2-methyl­imidazolato-κ^2^ *N*:*N*′)tris­(2-methyl­imidazole-κ*N*)dizinc(II)]

**DOI:** 10.1107/S1600536811015844

**Published:** 2011-05-07

**Authors:** Palanikumar Maniam, Norbert Stock

**Affiliations:** a Institut für Anorganische Chemie, Christian-Albrechts-Universität zu Kiel, Max-Eyth-Strasse 2, 24118 Kiel, Germany

## Abstract

Hydro­thermal reaction involving zinc nitrate hexa­hydrate, tris­odium benzene-1,3,5-tricarboxyl­ate (Na_3_BTC) and 2-methyl­imidazole (2-MeImH) yielded the title compound, [Zn_2_(C_9_H_3_O_6_)(C_4_H_5_N_2_)(C_4_H_6_N_2_)_3_]. In this mixed-ligand metal-organic compound, Zn^2+^ ions are coordinated by N atoms from 2-MeImH mol­ecules and (2-MeIm)^−^ ions, as well as by O atoms from (BTC)^3−^ ions. This results in two different distorted tetra­hedra, *viz.* ZnN_3_O and ZnN_2_O_2_. These tetra­hedra are inter­connected *via* (BTC)^3−^ ions and *N*:*N*′-bridging (2-MeIm)^−^ ions, thus forming a layered structure in the *bc* plane. Hydrogen bonds between the O atoms of carboxyl­ate ions and NH groups of 2-MeImH ligands link the layers into a three-dimensional structure.

## Related literature

For metal-organic frameworks, see: Li *et al.* (1999 ▶); Kitagawa *et al.* (2004 ▶); Stock (2010 ▶); Maniam *et al.* (2010 ▶). For related structures, see: Cheng *et al.* (2001 ▶); Zheng *et al.* (2010 ▶); Huang *et al.* (2006 ▶); Martins *et al.* (2010 ▶); Park *et al.* (2006 ▶).
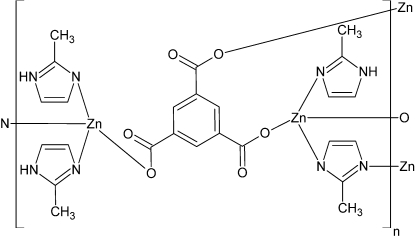


## Experimental

### 

#### Crystal data

[Zn_2_(C_9_H_3_O_6_)(C_4_H_5_N_2_)(C_4_H_6_N_2_)_3_]
*M* *_r_* = 665.28
Orthorhombic, 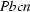

*a* = 18.9722 (6) Å
*b* = 18.2247 (4) Å
*c* = 16.5585 (4) Å
*V* = 5725.3 (3) Å^3^
*Z* = 8
Mo *K*α radiation
μ = 1.73 mm^−1^
*T* = 293 K
0.16 × 0.09 × 0.07 mm

#### Data collection

Stoe IPDS-1 diffractometer
Absorption correction: numerical (*X-RED* and *X-SHAPE*; Stoe & Cie, 2008 ▶) *T*_min_ = 0.684, *T*_max_ = 0.814
38494 measured reflections
7732 independent reflections
6222 reflections with *I* > 2σ(*I*)
*R*_int_ = 0.050

#### Refinement

*R*[*F* ^2^ > 2σ(*F* ^2^)] = 0.061
*wR*(*F* ^2^) = 0.143
*S* = 1.13
7732 reflections
370 parameters
H-atom parameters constrained
Δρ_max_ = 0.47 e Å^−3^
Δρ_min_ = −0.54 e Å^−3^

### 

Data collection: *X-AREA* (Stoe & Cie, 2008 ▶); cell refinement: *X-AREA*; data reduction: *X-AREA*; program(s) used to solve structure: *SHELXS97* (Sheldrick, 2008 ▶); program(s) used to refine structure: *SHELXL97* (Sheldrick, 2008 ▶); molecular graphics: *DIAMOND* (Brandenburg, 2010 ▶); software used to prepare material for publication: *XCIF* in *SHELXTL* (Sheldrick, 2008 ▶).

## Supplementary Material

Crystal structure: contains datablocks global, I. DOI: 10.1107/S1600536811015844/bt5531sup1.cif

Structure factors: contains datablocks I. DOI: 10.1107/S1600536811015844/bt5531Isup2.hkl

Additional supplementary materials:  crystallographic information; 3D view; checkCIF report

## Figures and Tables

**Table 1:** Selected geometric parameters (Å, °)

Zn1—O4	1.942 (3)
Zn2—O6	1.968 (3)
Zn2—O1^i^	1.976 (2)
Zn1—N2*H*^ii^	1.971 (3)
Zn1—N1*F*	1.998 (3)
Zn1—N1*G*	2.015 (4)
Zn2—N1*H*	1.992 (3)
Zn2—N1*E*	2.027 (3)

Symmetry codes: (i) 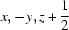; (ii) 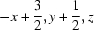.

**Table 2:** Hydrogen-bond geometry (Å, °)

*D*—H⋯*A*	*D*—H	H⋯*A*	*D*⋯*A*	*D*—H⋯*A*
N2*E*—H2*EN*⋯O3^iii^	0.86	2.06	2.912 (5)	169
N2*F*—H2*FN*⋯O2^iv^	0.86	1.84	2.693 (5)	172
N2*G*—H2*GN*⋯O5^v^	0.86	1.94	2.798 (5)	175

Symmetry codes: (iii) 
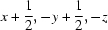
; (iv) 
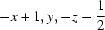
; (v) 
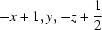
.

**Fig. 1.**
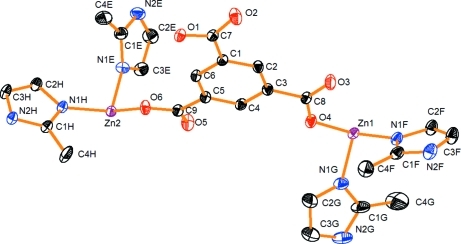
The asymmetric unit of (I), showing the atom-labelling scheme. Displacement ellipsoids are drawn at the 30% probability level and H atoms are omitted for clarity.

**Fig. 2.**
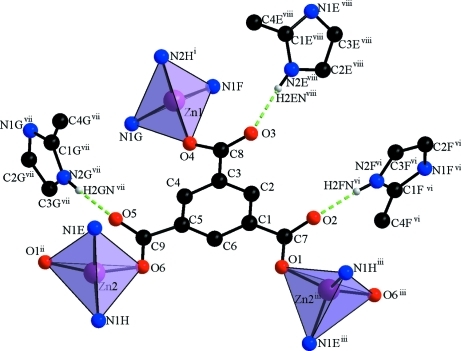
The tetrahedral coordination environment of the Zn1 and Zn2 ions. Green broken lines indicate the weak hydrogen bonds between the carboxylate groups of the (BTC)3- ions and H—N groups of the 2-MeImH ligands. [Symmetry codes: (i) -x + 3/2, y + 1/2, z; (ii) x, -y, z + 1/2; (iii) x, -y, z - 1/2; (iv) -x + 3/2, y - 1/2, z; (v) x + 1/2, -y + 1/2, -z; (vi) -x + 1, y, -z - 1/2; (vii) -x + 1, y, -z + 1/2; (viii) x - 1/2, -y + 1/2, -z.]

**Fig. 3.**
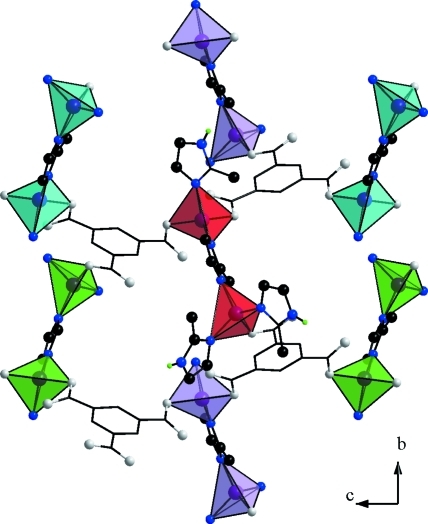
Ball-and-stick representation for (I) showing the interconnection of Zn-(2-MeIm)-Zn dimers (marked as differently colored polyhedra) by (BTC)3- ions and thus forming a layered arrangement in the b,c plane. Zn: purple, O: grey, N: blue, C: black and H: green. Hydrogen atoms of (BTC)3- are omitted for clarity.

**Fig. 4.**
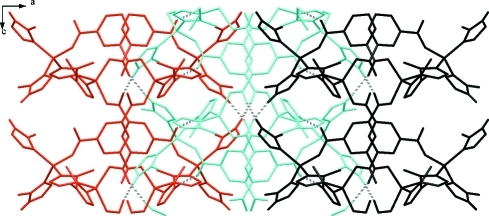
Layer packing diagram of compound (I). All atoms of one layer are presented by the same colour. H-bonds (see Fig. 2) between the layers are depicted by black broken lines.

## supplementary crystallographic information

### Comment

Metal-organic frameworks (MOF) are being investigated intensively, mainly for their high specific surface areas (Li *et al.*, 1999; Kitagawa *et al.*, 2004). In our workgroup, we are interested in using organic ligands containing multiple functional groups as the linkers for the MOFs. We employ high-throughput (HT) methods, which allow the rapid and systematic investigation of compound formation fields (Stock, 2010; Maniam *et al.*, 2010). HT-screening of various first row transition metal ions with trisodium benzene-1,3,5-tricarboxylate (Na_3_BTC) and 2-methylimidazole (2-MeImH) has yielded the colorless block crystals of (I). The asymmetric unit of compound (I) consists of two crystallographically independent Zn^2+^ ions, one fully deprotonated (BTC)^3-^, one 2-methylimidazolate (2-MeIm)^-^ ion and three 2-MeImH ligands (Fig. 1). The Zn^2+^ ions are tetrahedrally coordinated by oxygen atoms originating from (BTC)^3-^ and nitrogen atoms from (2-MeIm)^-^ and 2-MeImH. The Zn···O bond distances lie between 1.942 (3)–1.976 (2) Å which are slightly shorter than Zn···N bonds of 1.971 (3)–2.027 (3) Å. The bond angles in ZnN_3_O tetrahedra ranges between 100.42 (14)–116.61 (16)° while in ZnN_2_O_2_, the bond angles of 96.73 (13)–122.12 (11)° are observed (Tab. 1 & Tab. 2). It was also observed that Zn-(2-MeIm)^-^—Zn angle lies at 146.8 (1)° which is close to 145° angles in zeolitic imidazolate frameworks and zeolite structures (Park *et al.*., 2006). The C=O and C—O bonds in the carboxylate groups can be clearly distinguished from each other by their bond lengths of 1.229 (5)–1.236 (5) Å and 1.266 (4)–1.275 (4) Å, respectively. Weak hydrogen bonds in the 2.6 < *d*(O···H—N) < 3.0 Å range are observed between the O atoms of the carboxylate ions and N—H groups of the 2-MeImH ligands (Fig. 2).

By considering the ZnN_3_O and ZnN_2_O_2_ tetrahedra bridged by the (2-MeIm)^-^ as a Zn-(2-MeIm)-Zn dimer, this dimer is connected to three terminal 2-MeImH ligands and three (BTC)^3-^ ions. Each (BTC)^3-^ ion is then further connected to two Zn-(2-MeIm)-Zn dimers (Fig. 3) and layers in the *bc* plane are formed. Through extensive O···H—N hydrogen bonding, the layers are interconnected along the *a*-axis to form a dense three-dimensional crystal structure (Fig. 4, Tab. 2).

### Experimental

All reagents were of analytical grade (Aldrich and Fluka) and were used without further purification. High-throughput (HT) experiments in 300 ml Teflon-lined reactors yielded the crystals of compound (I). The reaction mixture consisted of zinc(II) nitrate hexahydrate (5.9 mg, 0.02 mmol), Na_3_(BTC) (2.76 mg, 0.01 mmol), 2-methylimidazole (4.11 mg, 0.05 mmol) and deionized water (200 ml). The mixture was heated in a 300 µl Teflon-lined high-throughput reactor at 423 K for 48 h (Stock, 2010). The mixture was cooled to room temperature over a period of 12 h and colourless plate-like crystals were obtained.

### Refinement

All H atoms were located in difference Fourier maps. Idealized values for the bond lengths (C—H = 0.93 Å and N—H = 0.86 Å) and angles were used and the H-atom parameters were refined using a riding model. The highest peak of 0.47 e Å^-3^ in the residual electron density map is located 0.82 Å from N1H and the deepest hole of 0.54 e Å^-3^ is located 0.69 Å from Zn1.

### Crystal data

**Table:**

[Zn_2_(C_9_H_3_O_6_)(C_4_H_5_N_2_)(C_4_H_6_N_2_)_3_]	*F*(000) = 2720
*M**_r_* = 665.28	*D*_x_ = 1.544 Mg m^−^^3^
Orthorhombic, *P**b**c**n*	Mo *K*α radiation, λ = 0.71073 Å
Hall symbol: -P 2n 2ab	Cell parameters from 40409 reflections
*a* = 18.9722 (6) Å	θ = 1.6–29.7°
*b* = 18.2247 (4) Å	µ = 1.73 mm^−^^1^
*c* = 16.5585 (4) Å	*T* = 293 K
*V* = 5725.3 (3) Å^3^	Block, colourless
*Z* = 8	0.16 × 0.09 × 0.07 mm

### Data collection

**Table:**

Stoe IPDS-1 diffractometer	7732 independent reflections
Radiation source: fine-focus sealed tube	6222 reflections with *I* > 2σ(*I*)
graphite	*R*_int_ = 0.050
φ scans	θ_max_ = 29.3°, θ_min_ = 1.6°
Absorption correction: numerical (*X-RED* and *X-SHAPE*; Stoe & Cie, 2008)	*h* = −26→26
*T*_min_ = 0.684, *T*_max_ = 0.814	*k* = −24→23
38494 measured reflections	*l* = −16→22

### Refinement

**Table:**

Refinement on *F*^2^	Primary atom site location: structure-invariant direct methods
Least-squares matrix: full	Secondary atom site location: difference Fourier map
*R*[*F*^2^ > 2σ(*F*^2^)] = 0.061	Hydrogen site location: inferred from neighbouring sites
*wR*(*F*^2^) = 0.143	H-atom parameters constrained
*S* = 1.13	*w* = 1/[σ^2^(*F*_o_^2^) + (0.0602*P*)^2^ + 8.9412*P*] where *P* = (*F*_o_^2^ + 2*F*_c_^2^)/3
7732 reflections	(Δ/σ)_max_ < 0.001
370 parameters	Δρ_max_ = 0.47 e Å^−^^3^
0 restraints	Δρ_min_ = −0.54 e Å^−^^3^

### Special details

**Table:**

Geometry. All e.s.d.'s (except the e.s.d. in the dihedral angle between two l.s. planes) are estimated using the full covariance matrix. The cell e.s.d.'s are taken into account individually in the estimation of e.s.d.'s in distances, angles and torsion angles; correlations between e.s.d.'s in cell parameters are only used when they are defined by crystal symmetry. An approximate (isotropic) treatment of cell e.s.d.'s is used for estimating e.s.d.'s involving l.s. planes.
Refinement. Refinement of *F*^2^ against ALL reflections. The weighted *R*-factor *wR* and goodness of fit *S* are based on *F*^2^, conventional *R*-factors *R* are based on *F*, with *F* set to zero for negative *F*^2^. The threshold expression of *F*^2^ > σ(*F*^2^) is used only for calculating *R*-factors(gt) *etc*. and is not relevant to the choice of reflections for refinement. *R*-factors based on *F*^2^ are statistically about twice as large as those based on *F*, and *R*- factors based on ALL data will be even larger.

### Fractional atomic coordinates and isotropic or equivalent isotropic displacement parameters (Å^2^)

**Table:**

	*x*	*y*	*z*	*U*_iso_*/*U*_eq_	
Zn1	0.45797 (2)	0.21003 (2)	0.06434 (3)	0.03012 (11)	
Zn2	0.86974 (2)	−0.02837 (2)	0.16875 (2)	0.02691 (11)	
O1	0.82544 (13)	0.04108 (16)	−0.22416 (15)	0.0317 (6)	
O2	0.75851 (19)	0.1297 (2)	−0.27156 (19)	0.0542 (9)	
O3	0.54340 (16)	0.20240 (19)	−0.1090 (2)	0.0482 (8)	
O4	0.53500 (14)	0.15579 (16)	0.01530 (17)	0.0372 (6)	
O5	0.73489 (17)	0.0422 (2)	0.14383 (17)	0.0461 (8)	
O6	0.81368 (14)	−0.01665 (16)	0.06956 (16)	0.0367 (6)	
C1	0.73604 (17)	0.0869 (2)	−0.1391 (2)	0.0262 (6)	
C2	0.67256 (19)	0.1251 (2)	−0.1327 (2)	0.0301 (7)	
H2A	0.6548	0.1500	−0.1773	0.036*	
C3	0.63563 (18)	0.1261 (2)	−0.0602 (2)	0.0299 (7)	
C4	0.66398 (18)	0.0910 (2)	0.0066 (2)	0.0301 (7)	
H4A	0.6397	0.0921	0.0554	0.036*	
C5	0.72805 (18)	0.0544 (2)	0.0019 (2)	0.0271 (7)	
C6	0.76310 (18)	0.0509 (2)	−0.0720 (2)	0.0276 (7)	
H6A	0.8048	0.0243	−0.0763	0.033*	
C7	0.77524 (18)	0.0865 (2)	−0.2179 (2)	0.0289 (7)	
C8	0.56601 (18)	0.1652 (2)	−0.0522 (2)	0.0302 (7)	
C9	0.76020 (19)	0.0245 (2)	0.0779 (2)	0.0296 (7)	
C1E	0.9480 (2)	0.1157 (3)	0.1420 (3)	0.0398 (9)	
C2E	0.9061 (3)	0.1880 (3)	0.2364 (3)	0.0512 (11)	
H2E	0.8966	0.2290	0.2680	0.061*	
C3E	0.8810 (2)	0.1201 (3)	0.2469 (3)	0.0423 (9)	
H3E	0.8505	0.1058	0.2879	0.051*	
C4E	0.9881 (3)	0.0916 (3)	0.0703 (3)	0.0579 (13)	
H4E1	1.0137	0.1325	0.0484	0.070*	
H4E2	0.9561	0.0730	0.0304	0.070*	
H4E3	1.0206	0.0537	0.0856	0.070*	
N1E	0.90718 (18)	0.07433 (19)	0.1879 (2)	0.0352 (7)	
N2E	0.9484 (2)	0.1849 (2)	0.1696 (3)	0.0471 (9)	
H2EN	0.9713	0.2210	0.1489	0.057*	
C1F	0.3558 (2)	0.1785 (2)	−0.0669 (3)	0.0396 (9)	
C2F	0.3373 (2)	0.2885 (3)	−0.0222 (3)	0.0427 (10)	
H2F	0.3412	0.3314	0.0079	0.051*	
C3F	0.2905 (2)	0.2767 (3)	−0.0822 (3)	0.0495 (11)	
H3F	0.2565	0.3093	−0.1010	0.059*	
C4F	0.3840 (3)	0.1032 (3)	−0.0801 (4)	0.0613 (15)	
H4F1	0.3585	0.0800	−0.1232	0.074*	
H4F2	0.3787	0.0749	−0.0315	0.074*	
H4F3	0.4330	0.1060	−0.0941	0.074*	
N1F	0.37830 (16)	0.22648 (19)	−0.0125 (2)	0.0353 (7)	
N2F	0.30319 (19)	0.2075 (2)	−0.1096 (3)	0.0479 (9)	
H2FN	0.2809	0.1861	−0.1482	0.057*	
C1G	0.3665 (3)	0.1373 (3)	0.1915 (4)	0.0604 (14)	
C2G	0.4502 (3)	0.0661 (3)	0.1528 (4)	0.0667 (16)	
H2G	0.4899	0.0485	0.1260	0.080*	
C3G	0.4119 (4)	0.0282 (4)	0.2072 (4)	0.0738 (17)	
H3G	0.4194	−0.0198	0.2241	0.089*	
C4G	0.3175 (5)	0.2000 (5)	0.1996 (7)	0.131 (4)	
H4G1	0.2818	0.1883	0.2386	0.157*	
H4G2	0.2958	0.2098	0.1484	0.157*	
H4G3	0.3431	0.2426	0.2172	0.157*	
N1G	0.4217 (2)	0.1347 (2)	0.1431 (2)	0.0444 (8)	
N2G	0.3604 (3)	0.0740 (3)	0.2321 (3)	0.0631 (13)	
H2GN	0.3290	0.0643	0.2680	0.076*	
C1H	0.94630 (18)	−0.1645 (2)	0.1130 (2)	0.0328 (8)	
C2H	1.01834 (19)	−0.0828 (2)	0.1579 (2)	0.0338 (8)	
H2H	1.0369	−0.0393	0.1782	0.041*	
C3H	1.05531 (18)	−0.1437 (2)	0.1393 (3)	0.0359 (8)	
H3H	1.1038	−0.1493	0.1452	0.043*	
C4H	0.8800 (2)	−0.2001 (3)	0.0853 (4)	0.0578 (15)	
H4H1	0.8900	−0.2490	0.0671	0.069*	
H4H2	0.8601	−0.1721	0.0418	0.069*	
H4H3	0.8470	−0.2020	0.1293	0.069*	
N1H	0.94875 (15)	−0.09609 (18)	0.14179 (19)	0.0302 (6)	
N2H	1.01007 (16)	−0.19593 (18)	0.1103 (2)	0.0324 (7)	

### Atomic displacement parameters (Å^2^)

**Table:**

	*U*^11^	*U*^22^	*U*^33^	*U*^12^	*U*^13^	*U*^23^
Zn1	0.02406 (18)	0.0339 (2)	0.0324 (2)	−0.00044 (16)	0.00318 (16)	−0.00303 (18)
Zn2	0.02578 (18)	0.0330 (2)	0.02195 (18)	0.00254 (16)	0.00150 (15)	0.00171 (16)
O1	0.0277 (11)	0.0457 (15)	0.0218 (11)	0.0058 (10)	0.0038 (9)	−0.0020 (11)
O2	0.064 (2)	0.067 (2)	0.0317 (15)	0.0266 (17)	0.0106 (14)	0.0175 (16)
O3	0.0392 (15)	0.059 (2)	0.0460 (17)	0.0201 (14)	0.0056 (14)	0.0122 (16)
O4	0.0305 (13)	0.0449 (15)	0.0361 (15)	0.0112 (11)	0.0091 (11)	0.0009 (12)
O5	0.0490 (17)	0.066 (2)	0.0232 (12)	0.0188 (15)	0.0029 (12)	0.0022 (14)
O6	0.0354 (13)	0.0493 (17)	0.0254 (12)	0.0162 (12)	−0.0044 (10)	0.0017 (12)
C1	0.0248 (15)	0.0310 (17)	0.0228 (15)	−0.0002 (13)	0.0014 (12)	−0.0024 (13)
C2	0.0306 (16)	0.0342 (18)	0.0253 (16)	0.0060 (14)	−0.0006 (13)	0.0027 (15)
C3	0.0293 (16)	0.0323 (17)	0.0281 (17)	0.0038 (13)	0.0033 (14)	0.0000 (14)
C4	0.0268 (15)	0.0383 (19)	0.0252 (16)	0.0017 (14)	0.0035 (13)	0.0017 (15)
C5	0.0274 (15)	0.0325 (17)	0.0215 (15)	0.0023 (13)	−0.0009 (12)	−0.0003 (13)
C6	0.0255 (15)	0.0323 (17)	0.0250 (16)	0.0041 (12)	−0.0006 (12)	−0.0012 (14)
C7	0.0283 (15)	0.0357 (18)	0.0228 (15)	0.0002 (13)	0.0002 (12)	0.0002 (14)
C8	0.0256 (15)	0.0348 (18)	0.0300 (18)	0.0031 (13)	0.0010 (13)	−0.0009 (15)
C9	0.0301 (16)	0.0369 (18)	0.0217 (15)	0.0030 (14)	−0.0010 (12)	0.0014 (14)
C1E	0.037 (2)	0.044 (2)	0.038 (2)	−0.0042 (17)	0.0001 (16)	0.0052 (18)
C2E	0.058 (3)	0.041 (2)	0.054 (3)	−0.005 (2)	−0.004 (2)	−0.009 (2)
C3E	0.042 (2)	0.044 (2)	0.041 (2)	−0.0027 (17)	0.0055 (17)	−0.0072 (19)
C4E	0.061 (3)	0.065 (3)	0.048 (3)	−0.002 (3)	0.019 (2)	0.014 (3)
N1E	0.0395 (17)	0.0330 (17)	0.0330 (17)	−0.0053 (13)	0.0025 (13)	−0.0001 (13)
N2E	0.049 (2)	0.0393 (19)	0.053 (2)	−0.0128 (16)	−0.0061 (18)	0.0047 (18)
C1F	0.0324 (18)	0.042 (2)	0.045 (2)	−0.0075 (16)	−0.0023 (16)	−0.0096 (19)
C2F	0.0330 (18)	0.043 (2)	0.052 (3)	0.0046 (16)	−0.0054 (17)	−0.016 (2)
C3F	0.037 (2)	0.057 (3)	0.055 (3)	0.0069 (19)	−0.0136 (19)	−0.014 (2)
C4F	0.058 (3)	0.040 (3)	0.086 (4)	−0.003 (2)	−0.010 (3)	−0.021 (3)
N1F	0.0261 (14)	0.0379 (17)	0.0420 (18)	−0.0006 (12)	−0.0046 (13)	−0.0075 (14)
N2F	0.0368 (17)	0.057 (2)	0.049 (2)	−0.0025 (16)	−0.0129 (16)	−0.0165 (19)
C1G	0.066 (3)	0.054 (3)	0.061 (3)	−0.016 (2)	0.034 (3)	−0.003 (3)
C2G	0.063 (3)	0.061 (3)	0.076 (4)	0.006 (3)	0.016 (3)	0.024 (3)
C3G	0.080 (4)	0.067 (4)	0.074 (4)	−0.005 (3)	0.008 (3)	0.028 (3)
C4G	0.130 (7)	0.093 (6)	0.169 (10)	0.021 (5)	0.116 (7)	0.016 (6)
N1G	0.0441 (19)	0.049 (2)	0.0405 (19)	−0.0053 (16)	0.0117 (16)	0.0037 (17)
N2G	0.072 (3)	0.070 (3)	0.047 (2)	−0.027 (2)	0.020 (2)	0.004 (2)
C1H	0.0244 (16)	0.039 (2)	0.0348 (19)	0.0002 (14)	0.0025 (14)	−0.0037 (16)
C2H	0.0288 (16)	0.041 (2)	0.0317 (19)	0.0012 (15)	−0.0039 (14)	−0.0053 (16)
C3H	0.0219 (15)	0.048 (2)	0.038 (2)	0.0040 (14)	−0.0044 (14)	−0.0122 (18)
C4H	0.0225 (18)	0.058 (3)	0.092 (4)	0.0010 (18)	0.000 (2)	−0.024 (3)
N1H	0.0244 (13)	0.0361 (16)	0.0302 (15)	0.0039 (12)	0.0007 (11)	−0.0044 (13)
N2H	0.0257 (14)	0.0363 (17)	0.0350 (16)	0.0034 (12)	0.0002 (12)	−0.0051 (14)

### Geometric parameters (Å, °)

**Table:**

Zn1—O4	1.942 (3)	N2E—H2EN	0.8600
Zn2—O6	1.968 (3)	C1F—N1F	1.325 (5)
Zn2—O1^i^	1.976 (2)	C1F—N2F	1.333 (6)
Zn1—N2H^ii^	1.971 (3)	C1F—C4F	1.490 (7)
Zn1—N1F	1.998 (3)	C2F—C3F	1.349 (6)
Zn1—N1G	2.015 (4)	C2F—N1F	1.381 (5)
Zn2—N1H	1.992 (3)	C2F—H2F	0.9300
Zn2—N1E	2.027 (3)	C3F—N2F	1.361 (6)
O1—C7	1.265 (4)	C3F—H3F	0.9300
O2—C7	1.228 (5)	C4F—H4F1	0.9600
O3—C8	1.236 (5)	C4F—H4F2	0.9600
O4—C8	1.274 (5)	C4F—H4F3	0.9600
O5—C9	1.236 (5)	N2F—H2FN	0.8600
O6—C9	1.269 (4)	C1G—N1G	1.319 (6)
C1—C6	1.388 (5)	C1G—N2G	1.340 (7)
C1—C2	1.395 (5)	C1G—C4G	1.480 (10)
C1—C7	1.503 (5)	C2G—C3G	1.348 (8)
C2—C3	1.390 (5)	C2G—N1G	1.372 (7)
C2—H2A	0.9300	C2G—H2G	0.9300
C3—C4	1.386 (5)	C3G—N2G	1.349 (8)
C3—C8	1.507 (5)	C3G—H3G	0.9300
C4—C5	1.389 (5)	C4G—H4G1	0.9600
C4—H4A	0.9300	C4G—H4G2	0.9600
C5—C6	1.394 (5)	C4G—H4G3	0.9600
C5—C9	1.501 (5)	N2G—H2GN	0.8600
C6—H6A	0.9300	C1H—N1H	1.336 (5)
C1E—N1E	1.321 (5)	C1H—N2H	1.339 (4)
C1E—N2E	1.340 (6)	C1H—C4H	1.488 (5)
C1E—C4E	1.477 (7)	C2H—C3H	1.348 (6)
C2E—C3E	1.338 (7)	C2H—N1H	1.369 (4)
C2E—N2E	1.368 (7)	C2H—H2H	0.9300
C2E—H2E	0.9300	C3H—N2H	1.368 (5)
C3E—N1E	1.377 (5)	C3H—H3H	0.9300
C3E—H3E	0.9300	C4H—H4H1	0.9600
C4E—H4E1	0.9600	C4H—H4H2	0.9600
C4E—H4E2	0.9600	C4H—H4H3	0.9600
C4E—H4E3	0.9600		
			
O4—Zn1—N2H^ii^	111.87 (13)	N1F—C1F—N2F	109.9 (4)
O4—Zn1—N1F	112.28 (13)	N1F—C1F—C4F	126.3 (4)
N2H^ii^—Zn1—N1F	110.38 (14)	N2F—C1F—C4F	123.8 (4)
O4—Zn1—N1G	100.42 (14)	C3F—C2F—N1F	109.0 (4)
N2H^ii^—Zn1—N1G	116.61 (16)	C3F—C2F—H2F	125.5
N1F—Zn1—N1G	104.81 (15)	N1F—C2F—H2F	125.5
O6—Zn2—O1^i^	122.12 (11)	C2F—C3F—N2F	106.1 (4)
O6—Zn2—N1H	106.66 (12)	C2F—C3F—H3F	127.0
O1^i^—Zn2—N1H	116.64 (13)	N2F—C3F—H3F	127.0
O6—Zn2—N1E	102.68 (13)	C1F—C4F—H4F1	109.5
O1^i^—Zn2—N1E	96.73 (13)	C1F—C4F—H4F2	109.5
N1H—Zn2—N1E	110.08 (13)	H4F1—C4F—H4F2	109.5
C7—O1—Zn2^iii^	118.0 (2)	C1F—C4F—H4F3	109.5
C8—O4—Zn1	130.2 (3)	H4F1—C4F—H4F3	109.5
C9—O6—Zn2	113.9 (2)	H4F2—C4F—H4F3	109.5
C6—C1—C2	119.6 (3)	C1F—N1F—C2F	106.2 (3)
C6—C1—C7	120.7 (3)	C1F—N1F—Zn1	125.3 (3)
C2—C1—C7	119.7 (3)	C2F—N1F—Zn1	128.5 (3)
C3—C2—C1	120.5 (3)	C1F—N2F—C3F	108.8 (4)
C3—C2—H2A	119.8	C1F—N2F—H2FN	125.6
C1—C2—H2A	119.8	C3F—N2F—H2FN	125.6
C4—C3—C2	119.2 (3)	N1G—C1G—N2G	110.0 (5)
C4—C3—C8	119.2 (3)	N1G—C1G—C4G	125.6 (5)
C2—C3—C8	121.6 (3)	N2G—C1G—C4G	124.4 (5)
C3—C4—C5	121.1 (3)	C3G—C2G—N1G	109.4 (6)
C3—C4—H4A	119.5	C3G—C2G—H2G	125.3
C5—C4—H4A	119.5	N1G—C2G—H2G	125.3
C4—C5—C6	119.3 (3)	N2G—C3G—C2G	106.1 (6)
C4—C5—C9	118.9 (3)	N2G—C3G—H3G	126.9
C6—C5—C9	121.7 (3)	C2G—C3G—H3G	126.9
C1—C6—C5	120.3 (3)	C1G—C4G—H4G1	109.5
C1—C6—H6A	119.9	C1G—C4G—H4G2	109.5
C5—C6—H6A	119.9	H4G1—C4G—H4G2	109.5
O2—C7—O1	123.7 (3)	C1G—C4G—H4G3	109.5
O2—C7—C1	119.8 (3)	H4G1—C4G—H4G3	109.5
O1—C7—C1	116.5 (3)	H4G2—C4G—H4G3	109.5
O3—C8—O4	125.5 (3)	C1G—N1G—C2G	105.9 (4)
O3—C8—C3	119.8 (3)	C1G—N1G—Zn1	129.8 (4)
O4—C8—C3	114.7 (3)	C2G—N1G—Zn1	124.2 (3)
O5—C9—O6	124.1 (3)	C1G—N2G—C3G	108.5 (4)
O5—C9—C5	119.2 (3)	C1G—N2G—H2GN	125.8
O6—C9—C5	116.7 (3)	C3G—N2G—H2GN	125.8
N1E—C1E—N2E	110.2 (4)	N1H—C1H—N2H	112.3 (3)
N1E—C1E—C4E	126.4 (4)	N1H—C1H—C4H	123.1 (3)
N2E—C1E—C4E	123.4 (4)	N2H—C1H—C4H	124.6 (4)
C3E—C2E—N2E	105.9 (4)	C3H—C2H—N1H	108.2 (3)
C3E—C2E—H2E	127.0	C3H—C2H—H2H	125.9
N2E—C2E—H2E	127.0	N1H—C2H—H2H	125.9
C2E—C3E—N1E	109.9 (4)	C2H—C3H—N2H	109.1 (3)
C2E—C3E—H3E	125.1	C2H—C3H—H3H	125.5
N1E—C3E—H3E	125.1	N2H—C3H—H3H	125.5
C1E—C4E—H4E1	109.5	C1H—C4H—H4H1	109.5
C1E—C4E—H4E2	109.5	C1H—C4H—H4H2	109.5
H4E1—C4E—H4E2	109.5	H4H1—C4H—H4H2	109.5
C1E—C4E—H4E3	109.5	C1H—C4H—H4H3	109.5
H4E1—C4E—H4E3	109.5	H4H1—C4H—H4H3	109.5
H4E2—C4E—H4E3	109.5	H4H2—C4H—H4H3	109.5
C1E—N1E—C3E	105.8 (4)	C1H—N1H—C2H	105.6 (3)
C1E—N1E—Zn2	130.0 (3)	C1H—N1H—Zn2	129.2 (2)
C3E—N1E—Zn2	122.9 (3)	C2H—N1H—Zn2	124.9 (3)
C1E—N2E—C2E	108.2 (4)	C1H—N2H—C3H	104.9 (3)
C1E—N2E—H2EN	125.9	C1H—N2H—Zn1^iv^	131.5 (3)
C2E—N2E—H2EN	125.9	C3H—N2H—Zn1^iv^	123.2 (2)
			
N2H^ii^—Zn1—O4—C8	−76.9 (4)	N1F—C2F—C3F—N2F	−0.3 (6)
N1F—Zn1—O4—C8	47.9 (4)	N2F—C1F—N1F—C2F	0.0 (5)
N1G—Zn1—O4—C8	158.8 (4)	C4F—C1F—N1F—C2F	179.9 (5)
O1^i^—Zn2—O6—C9	41.1 (3)	N2F—C1F—N1F—Zn1	−178.4 (3)
N1H—Zn2—O6—C9	178.8 (3)	C4F—C1F—N1F—Zn1	1.5 (7)
N1E—Zn2—O6—C9	−65.4 (3)	C3F—C2F—N1F—C1F	0.2 (5)
C6—C1—C2—C3	−1.3 (6)	C3F—C2F—N1F—Zn1	178.5 (3)
C7—C1—C2—C3	−180.0 (3)	O4—Zn1—N1F—C1F	37.9 (4)
C1—C2—C3—C4	2.5 (6)	N2H^ii^—Zn1—N1F—C1F	163.5 (3)
C1—C2—C3—C8	−178.4 (3)	N1G—Zn1—N1F—C1F	−70.2 (4)
C2—C3—C4—C5	−0.7 (6)	O4—Zn1—N1F—C2F	−140.1 (4)
C8—C3—C4—C5	−179.9 (3)	N2H^ii^—Zn1—N1F—C2F	−14.6 (4)
C3—C4—C5—C6	−2.2 (6)	N1G—Zn1—N1F—C2F	111.8 (4)
C3—C4—C5—C9	173.4 (4)	N1F—C1F—N2F—C3F	−0.2 (6)
C2—C1—C6—C5	−1.7 (5)	C4F—C1F—N2F—C3F	179.9 (5)
C7—C1—C6—C5	177.0 (3)	C2F—C3F—N2F—C1F	0.3 (6)
C4—C5—C6—C1	3.4 (5)	N1G—C2G—C3G—N2G	−1.1 (8)
C9—C5—C6—C1	−172.1 (3)	N2G—C1G—N1G—C2G	1.3 (7)
Zn2^iii^—O1—C7—O2	−9.8 (5)	C4G—C1G—N1G—C2G	−177.9 (8)
Zn2^iii^—O1—C7—C1	171.5 (2)	N2G—C1G—N1G—Zn1	178.2 (4)
C6—C1—C7—O2	−165.6 (4)	C4G—C1G—N1G—Zn1	−1.0 (11)
C2—C1—C7—O2	13.0 (6)	C3G—C2G—N1G—C1G	−0.1 (8)
C6—C1—C7—O1	13.2 (5)	C3G—C2G—N1G—Zn1	−177.2 (5)
C2—C1—C7—O1	−168.2 (3)	O4—Zn1—N1G—C1G	−175.7 (5)
Zn1—O4—C8—O3	−16.3 (6)	N2H^ii^—Zn1—N1G—C1G	63.3 (5)
Zn1—O4—C8—C3	164.1 (3)	N1F—Zn1—N1G—C1G	−59.1 (5)
C4—C3—C8—O3	173.2 (4)	O4—Zn1—N1G—C2G	0.7 (5)
C2—C3—C8—O3	−5.9 (6)	N2H^ii^—Zn1—N1G—C2G	−120.3 (5)
C4—C3—C8—O4	−7.2 (5)	N1F—Zn1—N1G—C2G	117.3 (5)
C2—C3—C8—O4	173.7 (4)	N1G—C1G—N2G—C3G	−2.0 (7)
Zn2—O6—C9—O5	−16.2 (5)	C4G—C1G—N2G—C3G	177.2 (8)
Zn2—O6—C9—C5	161.9 (3)	C2G—C3G—N2G—C1G	1.9 (8)
C4—C5—C9—O5	−10.7 (6)	N1H—C2H—C3H—N2H	0.7 (5)
C6—C5—C9—O5	164.7 (4)	N2H—C1H—N1H—C2H	0.4 (5)
C4—C5—C9—O6	171.0 (3)	C4H—C1H—N1H—C2H	−178.0 (4)
C6—C5—C9—O6	−13.5 (5)	N2H—C1H—N1H—Zn2	−173.3 (3)
N2E—C2E—C3E—N1E	0.2 (6)	C4H—C1H—N1H—Zn2	8.2 (6)
N2E—C1E—N1E—C3E	−0.1 (5)	C3H—C2H—N1H—C1H	−0.7 (4)
C4E—C1E—N1E—C3E	−179.9 (5)	C3H—C2H—N1H—Zn2	173.4 (3)
N2E—C1E—N1E—Zn2	167.5 (3)	O6—Zn2—N1H—C1H	−57.7 (4)
C4E—C1E—N1E—Zn2	−12.4 (7)	O1^i^—Zn2—N1H—C1H	82.7 (4)
C2E—C3E—N1E—C1E	−0.1 (5)	N1E—Zn2—N1H—C1H	−168.4 (3)
C2E—C3E—N1E—Zn2	−168.8 (3)	O6—Zn2—N1H—C2H	129.6 (3)
O6—Zn2—N1E—C1E	−60.7 (4)	O1^i^—Zn2—N1H—C2H	−89.9 (3)
O1^i^—Zn2—N1E—C1E	174.2 (4)	N1E—Zn2—N1H—C2H	18.9 (4)
N1H—Zn2—N1E—C1E	52.6 (4)	N1H—C1H—N2H—C3H	0.0 (5)
O6—Zn2—N1E—C3E	105.0 (3)	C4H—C1H—N2H—C3H	178.4 (5)
O1^i^—Zn2—N1E—C3E	−20.1 (4)	N1H—C1H—N2H—Zn1^iv^	−172.7 (3)
N1H—Zn2—N1E—C3E	−141.7 (3)	C4H—C1H—N2H—Zn1^iv^	5.7 (7)
N1E—C1E—N2E—C2E	0.2 (5)	C2H—C3H—N2H—C1H	−0.4 (5)
C4E—C1E—N2E—C2E	−179.9 (5)	C2H—C3H—N2H—Zn1^iv^	173.0 (3)
C3E—C2E—N2E—C1E	−0.2 (5)		

Symmetry codes: (i) *x*, −*y*, *z*+1/2; (ii) −*x*+3/2, *y*+1/2, *z*; (iii) *x*, −*y*, *z*−1/2; (iv) −*x*+3/2, *y*−1/2, *z*.

### Hydrogen-bond geometry (Å, °)

**Table:**

*D*—H···*A*	*D*—H	H···*A*	*D*···*A*	*D*—H···*A*
N2E—H2EN···O3^v^	0.86	2.06	2.912 (5)	169
N2F—H2FN···O2^vi^	0.86	1.84	2.693 (5)	172
N2G—H2GN···O5^vii^	0.86	1.94	2.798 (5)	175

Symmetry codes: (v) *x*+1/2, −*y*+1/2, −*z*; (vi) −*x*+1, *y*, −*z*−1/2; (vii) −*x*+1, *y*, −*z*+1/2.
